# Epiplastic microhabitats for epibenthic organisms: a new inland water frontier for diatoms

**DOI:** 10.1007/s11356-022-23335-8

**Published:** 2022-10-07

**Authors:** Davide Taurozzi, Giulia Cesarini, Massimiliano Scalici

**Affiliations:** Department of Sciences, University of Roma Tre, Viale G. Marconi 446, 00146 Rome, Italy

**Keywords:** Diatoms, Plastic colonization, Polystyrene, Polyethylene, Epiplastic community, Biofouling

## Abstract

Plastic pollution is widespread in each type of ecosystems. However, the colonization events of microorganisms on plastics seem to be neglected in inland waters. Therefore, in this study we analyze the possible colonization on the surface (hereafter epiplastic microhabitats) of two typology of plastic supports by diatom community. Specifically, we located 20 supports in expanded polystyrene and 20 in polyethylene terephthalate both floating and dipped (~ 1 m) in a central Italian shallow water pond, in order to evaluate the diachronic colonization of diatoms from November 2019 to August 2020. Our result showed the tendency in colonizing both epiplastic microhabitats without significant differences in number of species; additionally, depth does not appear to affect the number of species. As regard the temporal colonization, the number of species tends to increase over time from autumn-winter to spring-summer in both types of epiplastic microhabitats and depth. Instead, increase in dominance of some species over time has been demonstrated: only a few species keep a high number of individuals compared to the others; therefore, the number of individuals within the species is not uniformly distributed. These results suggest the tendency of diatom community to colonize plastic supports in lentic waters, and this evidence can be very important because artificial supports can increase the surface available for the settlement of the algae community with an increase of productivity and the colonization of new communities of different taxa. Further studies are mandatory to investigate the possible effects on the epiplastic community and the ecological implications in freshwater environments.

**Supplementary Information:**

The online version contains supplementary material available at 10.1007/s11356-022-23335-8.

## Introduction

The invention of plastic has given to the human being a material with almost unlimited uses, that is easy to produce, and has low cost, and for these reasons, its use has increased in recent decades in an excessive manner (Geyer et al. 2017). It is estimated that 368 million tons of plastic were produced worldwide in 2019 (PlasticsEurope 2020). To date, despite an increasing commitment to plastic disposal and recycling, it is estimated that around 79% of all plastic ever produced ended up in natural environments. Only 9% of plastic produced has been recycled, while 12% of all discarded plastic is disposed of in incinerators, generating an enormous number of toxic gases that are dispersed in the atmosphere (Geyer et al. 2017). Most of the plastics found in the oceans (80%) come from the continents, and the main causes of the plastic dumping are generally tourism, agriculture, wastewater, and fishing. Car tire abrasion also accounts for an important part of microplastics (< 5 mm) (Lanz and Gigon 2018). In the oceans, sea currents and winds carry the floating plastics offshore forming real islands called “plastic continents” where the concentration of plastic particles can reach 900,000 microplastics km^−2^ or even concentrations equal to 12,000 microplastics l^−1^ (Lanz and Gigon 2018).

As the amount of plastic debris has increased in different ecosystems, plastic pollution has started to be considered an environmental hazard of difficult management (van Emmerik and Schwarz 2020). The problem of plastic contamination is one of the greatest challenges that the discipline of environmental protection and research have been facing in recent decades. Plastics pollution is becoming a global issue that affects many habitats (Windsor et al. 2019; Cera et al. 2020; Cesarini et al. 2021), causing threats to aquatic life, ecosystem, and human health (van Emmerik and Schwarz 2020). However, the freshwater ecosystems are under investigated compared to the marine ones (Blettler et al. 2018). The most relevant impacts are ingestion (Gall and Thompson 2015; Bellasi et al. 2020), entrapment (Battisti et al. 2019; Lavers et al. 2020), chemical pollution (Besseling et al. 2013; Rochman et al. 2013), fragmentation (Weinstein et al. 2016; Hodgson et al. 2018), impacts on humans (Kosuth et al. 2018; Cook and Halden 2020). Moreover, plant communities, such as riparian habitats and mangrove forest trees, are impacted by plastic pollution, as these ecosystems function as traps or sinks (van Emmerik and Schwarz 2020; van Bijsterveldt et al. 2021; Cesarini and Scalici 2022). In addition, plastic debris can become solid surfaces for the transport of contaminants (Li et al. 2016), pathogens (Silva et al. 2019), and alien species (Gregory 2009). The plastic fragmentation can also generate smaller debris with different physical-chemical characteristics that cause a higher toxicity with organisms (Souza Machado et al. 2018). Moreover, as the plastic debris are hydrophobic, their surface enhances in few time the biofouling causing the following colonization by micro- and macroorganisms (Amaral-Zettler et al. 2021). The epiplastic community modifies the buoyancy, density, and fragmentation of the plastic litter colonized and causes sinking (Reisser et al. 2014). The colonizing plastic community is called “Plastisphere,” which consists in a unique, diverse, and complex microbial community that included diatoms, ciliates, and bacteria (Zettler et al. 2013). Among these microorganisms, diatoms are unicellular microalgae (10–500 μm) characterized by siliceous cell wall (frustule), unique photosynthetic pigments (diatoxanthin, diadinoxanthin, and fucoxanthin), and storage capacity of specific compounds (oil and chrysolaminarin) (ISPRA 2014).

Diatoms are both planktonic (phytoplankton) and benthic (phytobenthic) and populate all types of aquatic environments (i.e., fresh, brackish, and marine waters). In addition, diatoms are important primary producers at the base of food webs; it is estimated that they are responsible for the production of about 25% of all the oxygen produced on Earth (ISPRA 2014; Stevenson 2014). The diatom communities also have great importance as bioindicators for the water quality and are required by legislation, such as the Water Framework Directive (Marcheggiani et al. 2019). In marine habitat, diatoms were found to be the most abundant, widespread, and diverse group of plastic colonizers (Reisser et al. 2014; Davidov et al. 2020).

As regards the biofilm that forms on plastic supports in marine water, diatoms represent the most abundant type of eukaryotes (Caruso 2020). Previous studies show that the colonization of plastic substrates by diatoms in estuary zone occurs quite quickly, starting from 14 days after the insertion of the supports in water (Hudon and Bourget 1981). Carson et al. (2013) showed that the abundance of benthic diatoms from the North Pacific Gyre increases on supports with rough surfaces and in areas where there is a high concentration of plastics. Besides, some diatoms prefer certain polymers rather than others for colonization (Dudek et al. 2020).

The largest number of studies on this issue has been developed in marine waters, while only a few in running waters. In fact, this topic in inland waters and in particular in wetland habitats has been neglected to date. For this reason, we want to evaluate the colonization of diatom species in inland shallow water habitats depending on the type of plastic substrate. Sintered expanded polystyrene (PS) and polyethylene terephthalate (PE) were chosen as plastic supports as they are two of the most widely produced and used plastic materials (PlasticsEurope 2020). Specifically, we ask:

- How does the type of plastic polymers affect the diatom species assemblage?
- How does the depth of plastic polymers affect the diatom species assemblage?
- Does the diatom species assemblage on plastic substrates show a temporal trend?

## Material and methods

### Study area

The study was carried out in “Torre Flavia Natural Monument,” a protected wetland area located along the Tyrrhenian coast North of Rome (Ladispoli and Cerveteri Municipitatilies). The Torre Flavia Wetland is a Special Protection Zone (SPZ “Torre Flavia”; Code IT6030020), identified on the basis of European Bird Directive (147/2009/EC). In the study area, there is also a Site of Community Importance (“Secche di Torre Flavia,” S.I.C. IT6000009; Habitat Directive 92/43/CEE). Since the 1970s, the area has been progressively reclaimed, and this has led to the creation of a swamp in the rear dune area, made of many linked channels (Gustin 2021).

The study area presents some threats, such as the free use by visitators, the coastal erosion, the spread of alien species, the land use change, the waste accumulation (including plastic pollution) and the trampling of dunes. Some of these issues could become a threat for the many species present, like *Echinophora spinosa* (Linnaeus, 1753), *Cakile maritima* (Scopoli, 1772), *Pancratium maritimum* (Linnaeus, 1753) regarding vegetal component and *Bufo bufo* (Linnaeus, 1758), *Emys orbicularis* (Linnaeus, 1758), *Charadrius dubius* (Scopoli, 1786), or *Charadrius alexandrinus* (Linnaeus, 1758), regarding animal component (Gustin 2021).

### Experimental design

**Fig. 1:**
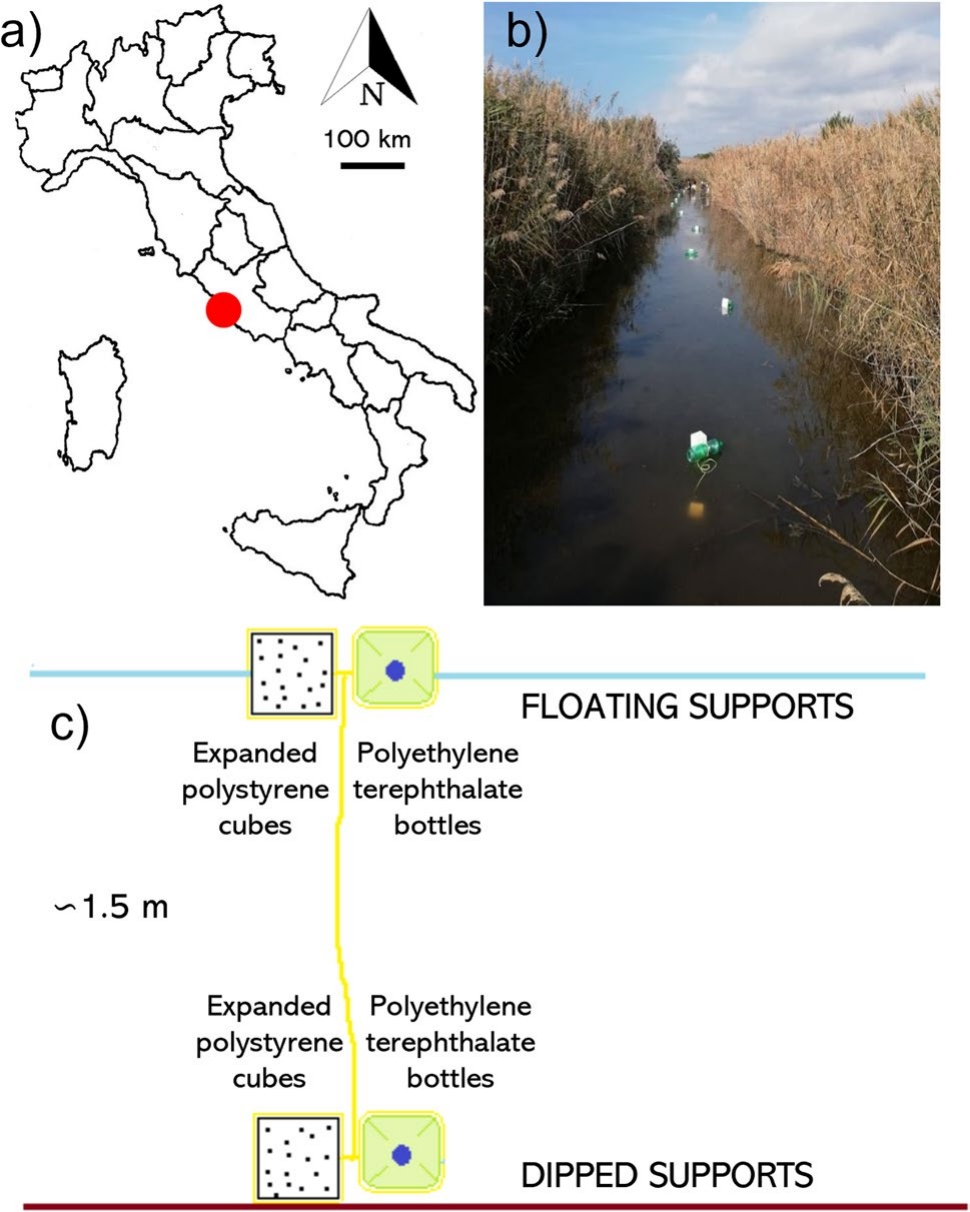
Experiment location and design. (**a**) The study area located in a shallow wetland in Latium Region, Central Italy. (**b**) The channel chosen for the support release and following sampling activities. (**c**) The plastic supports composed by expanded polystyrene cubes and polyethylene terephthalate bottles both floating on the water surface and dipped on the bottom (Paint). After the collection, the supports were brought to the bank, separated with a knife, and inserted into two different bags to avoid contamination of material between one unit and the others. The surfaces of each support were scraped using a commercial toothbrush, different for each of the four supports, to take samples of diatoms. Each toothbrush was then immersed in a 50 ml Falcon containing ethanol (70%) and water from the channel to preserve the samples

The sampling sites were placed within one of the numerous artificial canals that form a connecting network in the wetland area behind the dunes (Fig. 1). The entrance to the investigated area was fenced and prohibited for free use by visitors.

The study was conducted from November 2019 to August 2020. Ten plastic supports were created, each constituted by two PS structures and two PE structures (Fig. 1). PS is a rigid material, of low weight, derived from petroleum and composed of carbon, hydrogen, and 98% air, impermeable to water and free of chlorofluorocarbons (CFCs) and hydrochlorofluorocarbons (HCFCs). PE is a synthetic material belonging to the polyester family that is made with oil, natural gas, or vegetable raw materials and is 100% recyclable (de Macedo Vieira et al. 2020). Specifically, each support was made up of four units: two PS cubes and two PE bottles. One of the PE bottles was filled with sand to go to the bottom and bring with it a PS cube, while the remaining units of PS and PE were floating. Therefore, four typologies of epiplastic microhabitats were evaluated: floating and dipped polystyrene (fPS and dPS, respectively), and floating and dipped polyethylene (fPE and dPE). The supports were located about 3 m far from the others and were placed in water at the beginning of November 2019, taking care to arrange them equidistant respect to the banks of the canal. The samplings were conducted every 3 weeks (there was an interruption of sampling activities in the period from March to May due to the COVID-19 pandemic) until August 2020. During each sampling a plastic support composed of PS and PE was removed from the channel and analyzed (Fig. S2).

Samples were transported to the laboratory where oxidation of diatoms was carried out according to Italian protocol for inland water (ISPRA 2014). After homogenization of the samples, 7 ml was withdrawn from each sample, placed in a 15-ml centrifuge tube and centrifuged at 1500 rpm in an “Eppendorf 5810 R” centrifuge for 10 min, the supernatant was removed, and the remaining sample (about 0.5 ml) was placed in a 25-ml beaker. We proceeded through the oxidation method using hydrogen peroxide: 20 ml of hydrogen peroxide was added to the centrifuged sample, and the beaker was placed on a heating plate at 95 °C, waiting for all the substance organic to be oxidized (3–5 h). The beaker was removed from the plate when approximately 2–3 ml of substance remained, the contents were transferred to a centrifuge tube with distilled water, centrifuged at 1500 rpm, and the supernatant was removed. After having resuspended the contents with distilled water, two more decanting cycles were carried out and once all traces of hydrogen peroxide were eliminated, 2 ml of ethanol for the conservation of the samples was added. After the samples were oxidized, the permanent slides were prepared for the recognition of diatom species using Naphrax (high resin power of refraction) to fix the coverslip. The morphological identification of diatom species was conducted under a microscope at 100× magnification (Leica) and using taxonomic guides (Taylor et al. 2007; Ector and Hlúbiková 2010; ISPRA 2014; Bahls et al. 2018).

### Data analysis

Qualitative and quantitative analyses were carried out regarding the diatom community. The diatoms were classified at the species level, and following the temporal frequencies of the species most founded were calculated. The species most frequent were considered those that were found at least >50% during the samplings (5/10) for each type of plastic support.

A chi-square test was carried out to analyze the differences in the distribution of the number of individuals of the most abundant species in relation to the different types of substrates and to the different depths.

Then, we analyzed the number of diatom species in different supports and depths by the Kruskal-Wallis test (H).

To evaluate possible differences between the number of diatom species found on different types of supports (PS and PE) and different depths (floating and dipped), the *t* tests for paired data were performed.

Diversity indexes were calculated to test differences in the species distribution in function of the time by Mann-Kendall test. The data were transformed by log (x+1).

Specifically, the diversity indexes calculated are:

- Shannon diversity index, calculated as ${H}^{\prime }={\sum}_{j=1}^{\delta }{p}_j{\log}_e{p}_j$ (where *p*_*j*_ is the proportion of characters belonging to the *j*th type of letter in the string of interest);
- Simpson index, calculated as $\lambda ={\sum}_{i=1}^R{p}_i^2$ (where *R* is richness and *p*_*i*_ the proportional abundances);
- evenness, calculated as $J=\frac{H^{\prime }}{\log_2S}$ (where “H” is the value of the Shannon-Wiener diversity index and “S” is the number of species present in the given community);
- number of species (*N*).

The co-occurrence module was used to identify the presence of checkerboard pairs in the community, that is evidence of deterministic assembly rules. The C-score measures the average number of “checkerboard units” between all possible pairs of species; in a competitively structured community, there should be more checkerboard pairs of species than expected by chance.

Statistical tests were considered significant when *p* value was <0.05, while *p* value non-significant is reported as ns. All statistical analyses were computed in PAST 4.02 software (Hammer et al. 2001) and Ecosim 5.0 software (Gotelli and Entsminger 1999).

## Results

We found 97 species of diatoms overall on the 39 supports recovered (a PS support placed on the bottom was not found in the 10^th^ sampling). In Table 1 were shown the species with the highest temporal frequencies found on different supports and depths, i.e., those species found in at least 5 of the 10 total samplings.

**Table 1:** Frequencies of the most frequent species present respect the total number of supports on floating (fPS) and dipped polystyrene (dPS), on floating and dipped polyethylene (fPE and dPE)

Diatom species	fPS	dPS	fPE	dPE
*Achnanthes brevipes*	0.7		0.6	0.7
*Achnanthidium saprophilum*	0.7		0.6	
*Anomoeneis sphaerophora*	0.6		0.8	0.7
*Bacillaria paxillifera*	0.9	1	0.7	1
*Cyclotella comta*	0.6	0.7		0.7
*Fallacia pygmaea*				0.6
*Fragillaria rumpens*				0.8
*Gomphonema minutum*		0.6		
*Gomphonema zellense*	1	0.8	0.9	1
*Navicula criptocephala*	0.9	0.8	0.9	1
*Navicula tripunctata*	1	1	1	1
*Navicula veneta*	0.6			
*Nitzschia constricta*	0.9	1	0.9	1
*Nitzschia dubia*		0.7		0.8
*Nitzschia frustulum*	0.9	0.8	0.9	0.9
*Nitzschia hungarica*			0.7	0.6

Among the 97 species identified (Table S1), 34 were found in each of the 4 cases examined: *Achnantes brevipes, Achnanthidium saprophilum, Anomoeoneis sphaerophora, Bacillaria paxillifera, Cyclotella comta, Cyclotella meneghiniana, Cymbella compacta, Cymbella excisa, Cymbella parva, Encyonema minutum, Fallacia pygmaph veneta, Navicula cryptocephala, Navicula tripunctata, Navicula veneta, Nitzschia amphibia, Nitzschia angustatula, Nitzschia capitellata, Nitzschia communis, Nitzschia constricta, Nitzschia dissipata, Nitzschia dubia, Nitzschia filiformis, Nitzschia frustulum, Platessa hustedtii, Ulnaria ulna.*

In particular, were identified 8 species with an abundance higher than 3% respect to the total number of individuals (Table 2).

**Table 2:** Values of minimum (Min), median, and maximum (Max) of the species with the higher number of individuals (> 3%) respect to the total number of individuals

Diatom species	fPS			dPS			fPE			dPE		
	Min	Median	Max	Min	Median	Max	Min	Median	Max	Min	Median	Max
*Bacillaria paxillifera*	0	10	47	3	44	181	0	8	55	5	35	130
*Cyclotella comta*	0	4	72	0	4	29	0	1	68	0	9	86
*Gomphonema zellense*	1	6	37	0	3	83	0	11	62	1	12	232
*Navicula cryptocephala*	0	12	35	0	4	22	0	15	74	3	5	16
*Navicula tripunctata*	4	51	249	16	114	329	2	90	182	3	33	134
*Nitzschia constricta*	0	21	77	1	17	78	0	20	76	2	15	39
*Nitzschia filiformis*	0	2	44	0	0	77	0	4	86	0	0	68
*Nitzschia frustulum*	0	62	191	0	32	123	0	15	208	0	31	146

The chi-square test applied to the number of individuals of the most frequent species showed significant results in 40% of cases (species with no chi-square significant results in any of the four cases are not shown) (Table 3)

**Table 3:** Values of chi-square test (non-significative values are not shown)

Diatom species	Chi-square values			
	fPS vs dPS	fPEvs dPE	floating vs dipped	PS vs PE
*Bacillaria paxillifera*	205.29	210.78	415.87	-
*Gomphonema zellense*	7.33	184.57	169.24	204.23
*Navicula tripunctata*	-	138.83	30.78	137.71
*Nitzschia frustulum*	98.56	-	57.05	16.28

**Fig. 2:**
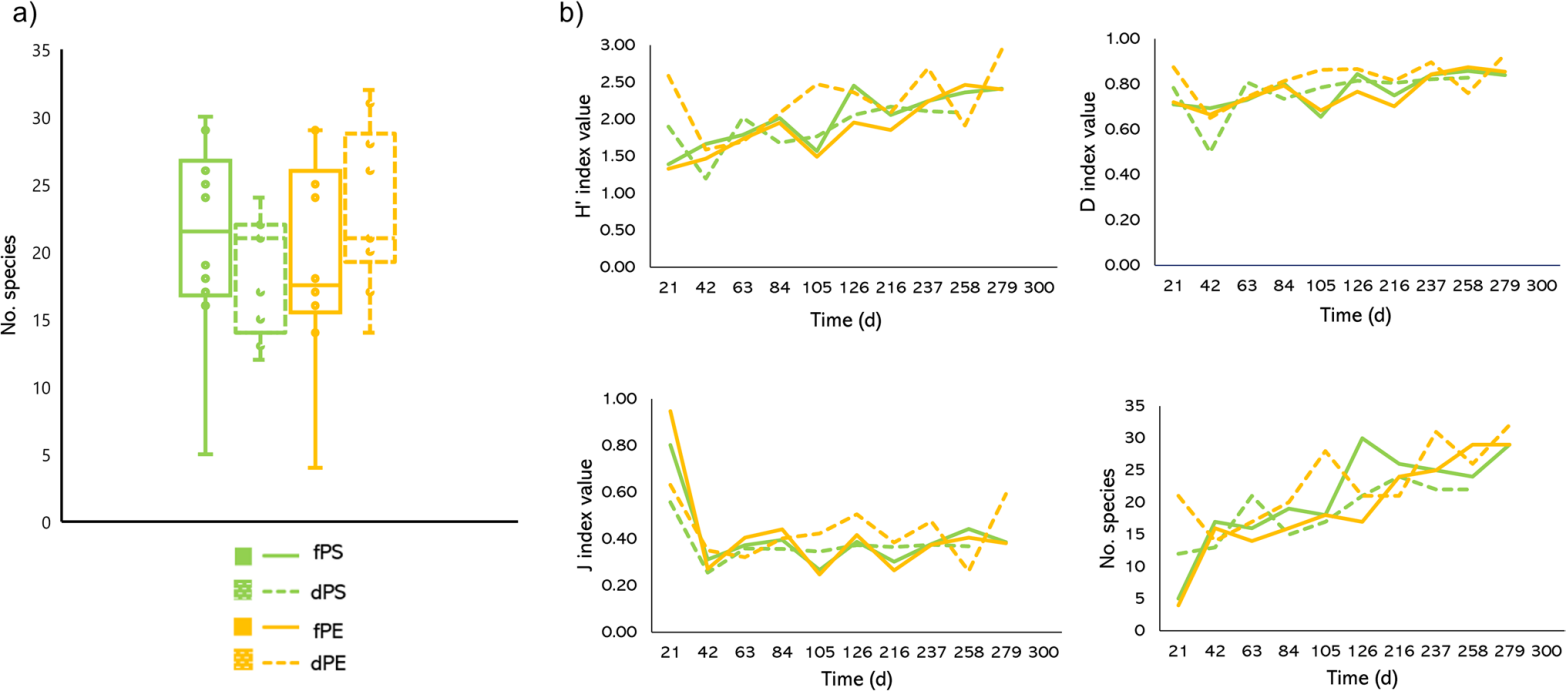
Values of the diatom communities that have colonized the plastic supports. (**a**) Number of diatom species found in each sampling on the 4 supports (the whiskers represent the variability outside the upper and lower quartiles). (**b**) Trends of the 4 diversity indices (*H*′ = Shannon, *λ* = Simpson, *J* = evenness, no. species = number of species), calculated for each sampling. fPS floating polystyrene, dPS dipped polystyrene, fPE floating polyethylene, fPE dipped polyethylene (Excel and Power Point)

The number of diatom species in different type of epiplastic microhabitats and depths were similar (Fig. 2), as confirmed by Kruskal-Wallis (*H*=0.81; *p* = ns)

The paired *t* test performed between the number of diatom species on PS and PE did not show significant differences in their distribution (*t* test = −1.20, *p* = ns). Moreover, the differences in the number of diatom species found on floating and dipped supports were not significance (*t* test = 0.97, *p* = ns). Therefore, the results highlighted that colonization of diatoms was independent of depth and substrate type.

**Fig. 3:**
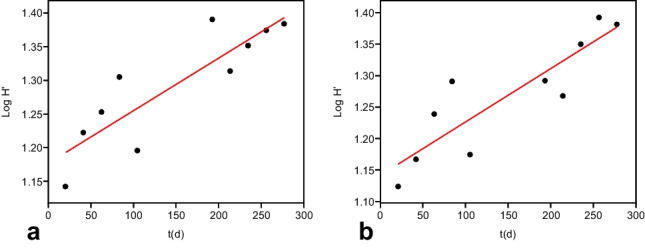
Plot of log transformed data showing the regression line as regards the Shannon index (*H*′) calculated for (**a**) floating polystyrene (fPs) and (**b**) floating polyethylene (fPE) supports (PAST)

The results of the diversity indices (Shannon index, Simpson index, evenness, number of species) calculated for each support are shown in Fig. 3.

The results obtained did not show a significant correlation with time for the evenness index in the four analyzed cases (*p* = ns), while showed a significant correlation with time for the Shannon index as regards the floating PS and PE supports (fPS: *R*^2^ = 0.756, *p* = 0.001; fPE: *R*^2^ = 0.77, *p* = 0.0007) (Fig. 3).

**Fig. 4:**
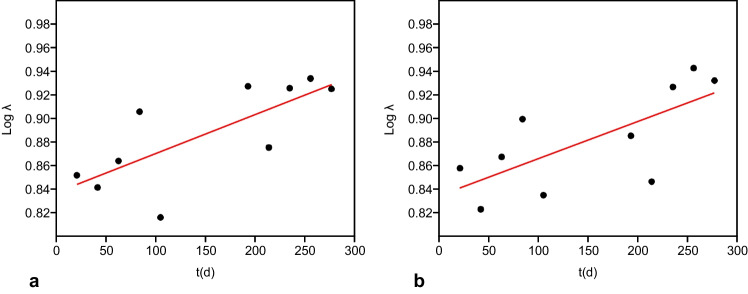
Plot of log transformed data showing the regression line as regards the Simpson index (*λ*) calculated for (**a**) floating polystyrene (fPs) and (**b**) floating polyethylene (fPE) supports (PAST)

The results also highlighted a significant correlation with time for the Simpson index as regards the floating PS and PE supports (fPS: *R*^2^ = 0.565, *p* = 0.012; fPE: *R*^2^ = 0.51, *p* = 0.020) (Fig. 4).

**Fig. 5:**
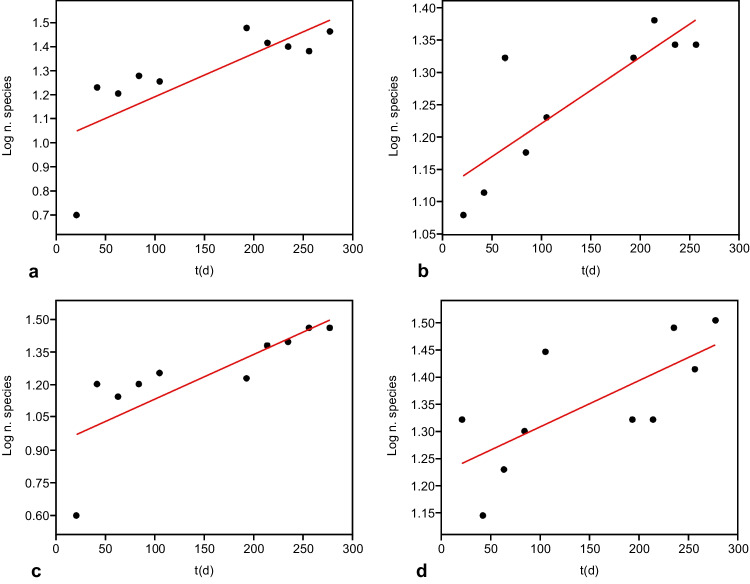
Plot of log transformed data showing the regression line as regards the number of species calculated for (**a**) floating polystyrene (fPS), (**b**) dipped polystyrene (fPS), (**c**) floating polyethylene (fPE), and (**d**) dipped polyethylene (dPE) supports (PAST)

Instead, a significant correlation with time for the number of species in all four investigated cases was found (fPS: *R*^2^ = 0.5823, *p* = 0.0102; dPS: *R*^2^ = 0.695, *p* = 0.0052; fPE: *R*^2^ = 0.619, *p* = 0.0068; dPE: *R*^2^ = 0.507, *p* = 0.02) (Fig. 5).

From the analysis of the co-occurrence, applied to the species found on plastic and polystyrene supports, regardless of depth, through presence/absence matrices, an observed index = 0.05434 was found, with a probability *p*_1_ (observed ≤ expected) = 0.89, and a probability *p*_1_′ (observed ≥ expected) = 0.11.

From the analysis of the co-occurrence, applied to the species found on the floating and dipped supports, regardless of the type of substrate, through presence/absence matrices, an observed index = 0.04897 was found, with a probability *p*_2_ (observed ≤ expected) = 0.95, and a probability *p*_2_′ (observed ≥ expected) = 0.05.

## Discussion

The results obtained in this study show a tendency of diatom species to colonize plastic artificial supports placed in lentic environments, contributing to investigate a topic little-known in these types of freshwater ecosystems. In particular, this study represent the first investigation of plastic colonization by freshwater microalgae in a wetland habitat. According to Lagarde et al. (2016), a solid surface of diverse materials placed in aquatic environment can be colonized by microorganisms such as bacteria, microalgae, and fungi. In marine environment, bacteria followed by microalgae resulted to be the first colonizers on plastic litter playing a key role and their colonization it is possible thanks to the production of exopolysaccharides, an extracellular polymeric substance (Khan et al. 2020). However, there are very few studies on the colonization of plastics by diatoms in freshwaters that can be taken as a comparison, but there are some studies on the effects of microplastics on other microalgae taxa conducted in laboratory.

The diatom colonization was observed on both types of plastic supports, PS and PE, and both on floating and dipped supports. The most frequently encountered diatom species are characteristic of waters with a medium-high trophic level, also populating environments from β to α meso-saprobic and medium-high electrolyte content (ISPRA 2014). These results are consistent with the ecological conditions present in the investigated area, that is a wetland located behind a dune area, affected by some phenomena, such as marine aerosol, which are usually found in the sandy stretch adjacent to the sea (Gustin 2021). The high trophic load is compatible with the phenomena of production and degradation of organic matter naturally present in wetland. Other studies highlighted the diatom colonization on PE and PS substrates in marine environments (Oberbeckmann et al. 2014, 2016; Reisser et al. 2014). Although, it is not clear to determine the influence of the type of plastic substrate and environmental factors in the colonization of diatoms (Nava and Leoni 2021).

The presence of the most abundant species found in the first samples, such as *Bacillaria paxillifera*, *Cyclotella comta*, *Cymbella compacta*, *Gomphonema pumilum*, *Gomphonema zellense*, *Navicula cryptocephala*, *Navicula tripunctata*, allowed the settlement of other species over time, increasing the number of species in the composition of diatomic community constant permanently. The number of species increased during the sampling period in all four cases of supports, as highlighted by the significant results of diversity indices. The *Navicula*, together with other genera, represent a genus very widespread and abundant on plastic debris in aquatic systems (Di Pippo et al. 2020; Kumar et al. 2017; Nava and Leoni 2021). The chi-square test has shown how the different distribution of individuals of the most frequent diatomic species depends both on the type of substrate and on the depth: in the floating supports, there is a greater light intensity than in the dipped supports, and the rough surface of the polystyrene, in addition to the various empty spaces present, can allow the adhesion and accumulation of diatoms. However, we hypothesize that in addition to only physical factors, ecological factors also intervene to model the different colonization by individuals of diatoms, such as the temperature, the turbidity of the water, or the salinity, depending on the different ecological and biotic characteristics of the species taken into consideration.

The increase in the number of species over time could be explained by the greater accumulation and replacement of nutrients on the surfaces of the plastic supports. In fact, the species that appear after the colonization of the first species do not have direct contact with the plastic surface but have a greater interaction with the biofilm formed (Elias and Banin 2012; Nava and Leoni 2021). Specifically, the values ​​of the Simpson index demonstrate dominance in all four cases, but only in the floating supports does a significant temporal trend appear, i.e., the dominance value tends to increase gradually. The values of the Shannon index, compared on the basis of time progression, show for the floating supports and the dipped PS a significant difference among the first half of the sampling period compared to second half.

By evaluating the correlation between the values of the indices and the time factor, the Simpson index and the Shannon index were found to have significant temporal trends for fPS and fPE supports. The number of individuals within species is not uniformly distributed, and this trend tends to increase over time, as shown by the significant correlation between the Simpson index and the time factor. High dominance values are common evidence in degraded environments. The type of substrate, therefore, does not affect the increase in dominance in the course of time of one or more species, while the depth at which the supports are found seems to do so. As regards the Shannon index, the results demonstrate a significant temporal trend, i.e., show, in the light of the results of the other indices, an addiction to the increase in the number of species rather than on variations in the evenness. The differences that emerged between dipped and floating supports can be linked to the fact that in the supports on the water surface there is a greater light intensity than in the dipped supports, and this could in some way allow the increase in the dominance of some species rather than others over time. In addition to this, it should be considered that the floating supports are more exposed to atmospheric phenomena, including wind, which can favor the transport, adhesion, and exchange of nutrients than the ones on the bottom, but with differences between the types of substrate.

From our analyses emerges that the diatomic assemblage founded is not a community structured on competition: the observed C-score, lower than expected by chance, demonstrates how the distribution of the diatom species traced in our studies depends on external factors (we hypothesize physical and chemicals parameters) and not by inter-specific interactions. Anyway, it is evident that the diatom community colonizes plastic substrates regardless of the type of polymer in a relatively short time; therefore, we hypothesize that any plastic waste can be colonized by diatoms. It is important to highlight that a similar condition could be temporary because, according to Khan et al. (2020), the litter sinking capability is enhanced by biofilms (exopolysaccharides) formed on their surfaces that increases the weight, so the floating supports could sink. Diatoms are important and abundant component of the biofilm biodiversity that colonizes the plastic in aquatic systems (Caruso 2020; Di Pippo et al. 2020; Zhao et al. 2021). In addition to increasing plastic density (Khan et al. 2020; Chen et al. 2019), the biofouling can modify other plastic properties such as alteration in adsorption capability of environmental pollutants, UV-protection and delay the phenomenon of degradation. Moreover, the presence of biofilm on plastic makes them more attractive to be ingested by other organisms and consequently alters the entire trophic chain (Nava and Leoni 2021).

The findings emerged by this study can have positive but also negative implications on the ecosystem. In fact, plastic can represent a new habitat for diatom and form as substrate for the colonization of primary producers, supporting the biodiversity. However, the fragmentation of plastic in smaller size and the exposure to environmental factors and pollutants can modify the chemical-physical properties of plastics. Some studies highlight that the presence of plastic can reduce the pollutants available in the environment. In fact, plastics could adsorb hydrophobic organic contaminants from contaminated water and reduce the concentrations available to microalgae, which could be the mechanism of minimizing toxicity when organic contaminants are exposed to microalgae in combination with microplastics (Guo et al. 2020). According to Yang et al. (2020), all types of microplastics and nonylphenol exerted significant inhibitory effects on microalgae growth, and their toxicities were antagonistic. The combined toxicity showed a positive effect by microplastics reducing the toxicity of nonylphenol to algae due to microplastic adsorption behavior, specially the smaller sizes (Yang et al. 2020). Garrido et al. (2019) have shown that microplastics change the toxicity of the Chlorpyrifoson pesticide on the microalgae growth reducing its toxicity due to the adsorption of pesticide on the microplastic surface that makes it less bio-available to the algae. Moreover, it is important to evaluate the size of microplastics used during the tests as their dimension must allow to penetrate the microalgal cells and cause possible damages (Garrido et al. 2019).

On the other hand, Baudrimont et al. (2020) highlighted that polyethylene collected from the field causes growth inhibition of *Scenedemus subspicatus* for freshwater microalgae at all the exposure concentrations tested due to the possible presence of metals. Moreover, Guschina et al. (2020) showed that polystyrene microplastics affect two major compounds (i.e., waxes and steryl esters) of the cell wall in the freshwater microalgae *Isochrysis galbana*, reducing their concentration. In particular, they suppose that polystyrene microplastics could be absorbed by the cells of the microalgae and may be accumulated into the cell wall causing possible biomagnification through trophic transfer from primary producers to consumers (Guschina et al. 2020). Therefore, the presence of plastic in the environment can represent a resource as new habitat and substrate for primary producers and reduce the concentration of other pollutants or can be a threat for biota causing different impacts. Further studies are mandatory to better understand all possible effects due to the presence of plastic in wetland ecosystems.

## Conclusions

Given the lack of knowledge about the microorganisms that colonize plastics in freshwaters, this study is a valuable contribution to begin to fill this gap. In summary, this study shows the tendency of diatoms to colonize plastic supports artificially placed in a wetland as epiplastic microhabitats. Numerous studies face the problem of plastic pollution in marine environments, but the data presented here represent the first evidence of colonization by diatoms on plastic supports artificially placed in a wetland. This phenomenon could have very interesting implications from the point of view of increasing the productivity of the ecosystem: artificial supports can increase the surface available for the settlement of the algae community which could lead to an increase in productivity in general. Furthermore, the presence of algae compounds could favor the establishment of an animal community. This perspective could pave the way for new studies to try to understand the impacts of the ingestion of epiplastic diatoms on consumers. Therefore, further studies should be conducted to better understand the influence of plastic polymers and environmental conditions in colonization processes and deepen the ecological implication of the diatom and plastic interactions.

## Supplementary information

The online version contains supplementary material available at 10.1007/s11356-022-23335-8.

ESM 1 (PDF 255 kb)

## Data Availability

The datasets used and/or analyzed during the current study are available from the corresponding author on reasonable request.
