# Supplementary material for: Epiplastic microhabitats for epibenthic organisms: a new inland water frontier for diatoms
Source: Environ Sci Pollut Res Int. 2022 Oct 7;30(7):17984–93. doi: 10.1007/s11356-022-23335-8 (PMC9540040; doi:10.1007/s11356-022-23335-8)

Supplementary Information

Epiplastic microhabitats for epibenthic organisms: a new inland water frontier for diatoms

Davide Taurozzi[1], Giulia Cesarini[1*] and Massimiliano Scalici[1]

[1] Department of Sciences, University of Roma Tre, Viale G. Marconi 446, 00146 Rome, Italy

[*]Corresponding author. Email address: giulia.cesarini@uniroma3.it.

Submitted to *Environmental Science and Pollution Research*

Table S1 Species found on floating and dipped polystyrene (fPS, dPS), and of floating and dipped polyethylene (fPE, dPE), abundance, and relative frequency in bracket

Species	fPS	dPS	fPE	dPE
Achnanthes brevipes	202 (0.70)	4 (0.30)	68 (0.60)	39 (0.70)
Achnanthidium minutissimum	40 (0.20)	4 (0.10)	193 (0.20)	
Achnanthidium saprophilum	83 (0.70)	37 (5)	36 (0.60)	35 (0.50)
Achnanthidium subatomus	15 (0.10)			
Achnantidium exiguum	1 (0.10)			
Amphora coffeaeformis	1 (0.10)		1 (0.10)	
Amphora merdionalis			1 (0.10)	
Amphora vetula	2 (0.10)	1 (0.10)		5 (0.10)
Anomoeneis sphaerophora	38 (0.60)	14 (0.50)	78 (0.80)	77 (0.70)
Bacillaria paxillifera	159 (0.90)	537 (0.90)	141 (0.70)	512 (100)
Caloneis amphisbaena	1 (0.10)			12 (0.20)
Caloneis bacillum	2 (0.10)			
Cocconeis neodiminuta				1 (0.10)
Cocconeis pediculus			2 (0.10)	
Craticola buderi	5 (0.30)	8 (0.30)	5 (0.30)	30 (0.30)
Craticula cuspidata				2 (0.10)
Cyclotella comta	148 (0.60)	73 (0.30)	97 (0.50)	214 (0.70)
Cyclotella meneghiniana	61 (0.40)	45 (0.30)	51 (0.40)	74 (0.40)
Cymatopleura solea	1 (0.10)			
Cymbella compacta	47 (0.20)	6 (0.20)	28 (0.30)	14 (0.10)
Cymbella excisa	6 (0.30)	6 (0.20)	17 (0.20)	17 (0.40)
Cymbella parva	15 (0.50)	6 (0.40)	13 (0.30)	6 (0.20)
Diatoma ehrenbergii		1 (0.10)	4 (0.20)	
Diatoma moniliformis	1 (0.10)			1 (0.10)
Diatoma tenuis	1 (0.10)		1 (0.10)	
Diatoma vulgaris				1 (0.10)
Diploneis parma	2 (0.20)		1 (0.10)	7 (0.20)
Encyonema minutum	37 (0.20)	32 (0.20)	12 (0.10)	8 (0.10)

Encyonema prostratum				1 (0.10)
Encyonema silesiacum				4 (0.10)
Epithemia adnata	4 (0.30)			4 (0.40)
Ephitemia sorex				1 (0.10)
Eucocconeis flexella	1 (0.10)	2 (0.20)		2 (0.20)
Eunotia minor	4 (0.30)	1 (0.10)		10 (0.30)
Eunotia pectinalis			1 (0.10)	1 (0.10)
Fallacia pygmaea	9 (0.50)	4 (0.30)	4 (0.20)	40 (0.60)
Fragilaria gracilis		10 (0.10)	5 (0.20)	9 (0.10)
Fragillaria pectinalis		4 (0.20)		
Fragillaria pinnata	1 (0.10)	2 (0.10)		
Fragillaria rumpens	37 (0.50)	21 (0.30)	22 (0.40)	109 (0.80)
Gomphonema augur			1 (0.10)	
Gomphonema elegantissimum		2 (0.10)	3 (0.10)	
Gomphonema italicum			1 (0.10)	1 (0.10)
Gomphonema micropus			1 (0.10)	
Gomphonema minutum	59 (0.30)	15 (0.60)	162 (0.50)	10 (0.40)
Gomphonema pumilum	21 (0.40)	17 (0.30)	11 (0.40)	67 (0.50)
Gomphonema subclavatum			3 (0.10)	2 (0.10)
Gomphonema zellense	110 (100)	154 (0.70)	174 (0.90)	536 (100)
Gyrosigma sciotoense	3 (0.30)	2 (0.20)		5 (0.40)
Halamphora normannii	1 (0.10)			
Halamphora veneta	74 (0.40)	113 (0.40)	71 (0.40)	40 (0.40)
Hantzschia amhilepta	1 (0.10)	1 (0.10)	1 (0.10)	
Navicula antonii	5 (0.20)	8 (0.10)	4 (0.20)	
Navicula cari				3 (0.10)
Navicula communis	2 (0.10)			
Navicula cryptocephala	137 (0.90)	59 (0.70)	178 (0.90)	69 (100)
Navicula cryptotenella	1 (0.10)		3 (0.10)	4 (0.10)
Navicula gregaria		10 (0.20)		4 (0.10)
Navicula recens	5 (0.10)			2 (0.10)
Navicula rostellata			1 (0.10)	
Navicula simulata		2 (0.10)		
Navicula tripunctata	1002 (100)	1116 (0.90)	932 (100)	488 (100)
Navicula veneta	29 (0.60)	9 (0.30)	5 (0.10)	19 (0.50)
Nitzschia amphibia	2 (0.10)	1 (0.10)	2 (0.20)	2 (0.20)
Nitzschia angustatula	10 (0.30)	32 (0.50)	27 (0.30)	13 (0.40)
Nitzschia capitellata	2 (0.20)		6 (0.40)	2 (0.20)
Nitzschia communis	11 (0.30)	5 (0.40)	5 (0.20)	9 (0.30)
Nitzschia constricta	267 (0.90)	271 (0.90)	274 (0.90)	156 (100)

Navicula cuspidata	1 (0.10)			
Nitzschia dissipata	7 (0.20)	13 (0.30)	15 (0.20)	3 (0.20)
Nitzschia dubia	13 (0.40)	20 (0.60)	12 (0.30)	84 (0.80)
Nitzschia filiformis	119 (0.60)	135 (0.40)	224 (0.50)	132 (0.40)
Nitzschia frustulum	809 (0.90)	456 (0.70)	541 (0.90)	529 (0.90)
Nitzschia hungarica	52 (0.50)	9 (0.40)	49 (0.70)	21 (0.60)
Nitzschia levidensis	1 (0.10)	1 (0.10)		5 (0.20)
Nitzschia linearis	27 (0.40)	93 (0.30)	18 (0.40)	117 (0.50)
Nitzschia microcephala	8 (0.30)	5 (0.10)	17 (0.20)	
Nitzschia palea		2 (0.20)	1 (0.10)	2 (0.10)
Nitzschia pusilla				1 (0.10)
Nitzschia recta	4 (0.10)		1 (0.10)	7 (0.30)
Nitzschia scalpelliformis	2 (0.10)		2 (0.20)	
Nitzschia sigma	10 (0.10)		3 (0.20)	1 (0.10)
Nitzschia umbonata			1 (0.10)	8 (0.10)
Planothidium frequentissimum	20 (0.20)	31 (0.40)	28 (0.30)	38 (0.50)
Planothidium rostratum	1 (0.10)	1 (0.10)	1 (0.10)	3 (0.30)
Platessa hustedtii	3 (0.10)	3 (0.10)	1 (0.10)	165 (0.10)
Reimeria sinuata				2 (0.10)
Rophalodia gibba			1 (0.10)	1 (0.10)
Rophalodia musculus			121 (0.10)	
Sellaphora bacillum				5 (0.10)
Surirella brebissonii			1 (0.10)	
Surirella brightvellii	3 (0.10)	1 (0.10)		1 (0.10)
Surirella ovalis	1 (0.10)	4 (0.10)	3 (0.20)	2 (0.20)
Tabularia fasciculata	4 (0.10)			
Ulnaria acus	1 (0.10)		1 (0.10)	11 (0.30)
Ulnaria biceps			1 (0.10)	
Ulnaria ulna	24 (0.50)	8 (0.20)	29 (0.40)	25 (0.20)

Table S2 Scheme of the experimental design for the sampling of diatom epiplastic community.

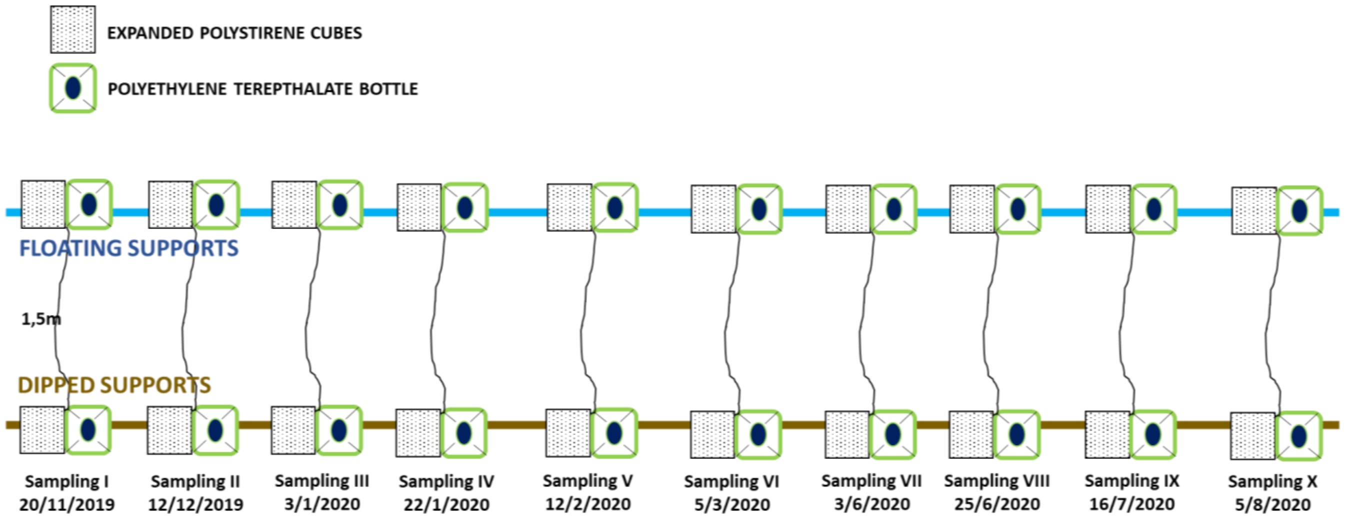

Supplement: Supplementary file 1 — (PDF 255 kb) [file 11356_2022_23335_MOESM1_ESM.pdf]
